# A qualitative study of women’s and health providers’ attitudes and acceptability of mistreatment during childbirth in health facilities in Guinea

**DOI:** 10.1186/s12978-016-0262-5

**Published:** 2017-01-13

**Authors:** Mamadou Diouldé Balde, Abou Bangoura, Boubacar Alpha Diallo, Oumar Sall, Habibata Balde, Aïssatou Sona Niakate, Joshua P. Vogel, Meghan A. Bohren

**Affiliations:** 1 Cellule de recherche en santé de la reproduction en Guinée (CERREGUI), University National Hospital-Donka, Conakry, Guinea; 2 Faculté de médecine, pharmacie et odontostomatologie, Université G.A. Nasser de Conakry, Conakry, Guinea; 3 Département de sociologie, Université Sonfonia, Conakry, Guinea; 4 UNDP/UNFPA/UNICEF/WHO/World Bank Special Programme of Research, Development and Research Training in Human Reproduction (HRP), Department of Reproductive Health and Research, World Health Organization, Avenue Appia 20, 1211 Geneva, Switzerland

**Keywords:** Maternal health, Mistreatment, Disrespect and abuse, Qualitative research, Violence, Quality of care, Guinea, Childbirth

## Abstract

**Background:**

Reducing maternal morbidity and mortality remains a key health challenge in Guinea. Anecdotal evidence suggests that women in Guinea are subjected to mistreatment during childbirth in health facilities, but limited research exists on this topic. This study was conducted to better understand the social norms and the acceptability of four scenarios of mistreatment during childbirth, from the perspectives of women and service providers.

**Methods:**

This study used qualitative methods including in-depth interviews (IDIs) and focus group discussions (FGDs) with women of reproductive age, midwives, nurses and doctors. This study was conducted in one urban area (Mamou) and one peri-urban area (Pita) in Guinea. Participants were presented with four scenarios of mistreatment during childbirth, including a provider: (1) slapping a woman; (2) verbally abusing a woman; (3) refusing to help a woman; and (4) forcing a woman to give birth on the floor. Data were collected in local languages (Pular and Malinké) and French, and transcribed and analyzed in French. We used a thematic analysis approach and manually coded the data using a codebook developed for the project.

**Results:**

A total of 40 IDIs and eight FGDs were conducted with women of reproductive age, 5 IDIs with doctors, and 13 IDIs with midwives. Most women were not accepting of any of the scenarios, unless the action was perceived to be used to save the life of the mother or child. However, they perceived a woman’s disobedience and uncooperativeness to contribute to her poor treatment. Women reacted to this mistreatment by accepting poor treatment, refusal to use the same hospital, revenge against the provider or complaints to hospital management. Service providers were accepting of mistreatment when women were disobedient, uncooperative, or to save the life of the baby.

**Conclusions:**

This is the first known study on mistreatment of women during childbirth to be conducted in Guinea. Both women and service providers were accepting of mistreatment during childbirth under certain conditions. Any approach to preventing and eliminating mistreatment during childbirth must consider these important contextual and social norms and develop a comprehensive intervention that addresses root causes. Further research is needed on how to measure mistreatment during childbirth in Guinea.

## Plain English summary

Global research evidence suggests that women may be mistreated during childbirth; for example this may include slapping, pinching, verbal abuse, and discrimination. In Guinea, a country in West Africa, anecdotal evidence suggests that women are mistreated during childbirth in hospitals. In this study, we used qualitative methods (in-depth interviews and focus group discussions) to explore how attitudes and social norms influence how women are treated during childbirth, from the perspectives of women and healthcare providers in Guinea. Research participants were presented with four scenarios detailing forms of mistreatment of women during childbirth: (1) a provider pinching or slapping a woman in labor; (2) a provider yelling or shouting at a woman in labor; (3) a provider refusing to help a woman in labor; and (4) forcing a woman to deliver on the floor of the hospital. Participants were asked if the scenario was acceptable, under what circumstances it would be acceptable, and how they would feel if it happened to them. Most women were not accepting of any of the scenarios, unless the action was perceived to be used to save the life of the mother or child. Service providers were accepting of mistreatment when women were disobedient, uncooperative, or to save the life of the baby. This study suggests that women in Guinea experience mistreatment during childbirth, and that they may be accepting of this mistreatment under certain circumstances. In order to prevent mistreatment during childbirth from happening in Guinea, these social norms must be taken into account.

## Background

Pregnancy and childbirth continue to place women at a substantial risk of mortality and morbidity, particularly in low- and middle-income countries (LMICs) [1]. Maternal mortality is defined as the death of a woman during pregnancy or in the 42 days after the termination of pregnancy from pregnancy-related causes [1]. A report from the World Health Organization estimates that 303,000 maternal deaths occurred in 2015, despite global efforts to reduce maternal mortality as part of the Millennium Development Goals (MDGs) [1]. Maternal mortality in LMICs is approximately 20 times higher than high-income countries, and Sub-Saharan Africa accounts for almost two-thirds of the global burden of maternal mortality [1].

Improving quality of care during childbirth is an integral component of improving maternal health [2]. Quality of care includes both the *provision* of care (such as evidence-based clinical practices, information systems, and referral systems), as well as the *experience* of care (such as respect, communication and emotional support) [2]. Quality care should be implemented with motivated healthcare providers in a health facility with adequate physical resources [2]. However, efforts to improve quality of care have historically focused on improving the provision of care, and research on how to improve women’s experiences of childbirth care has been largely neglected.

Global evidence suggests that women may be mistreated during childbirth [3]. For example this may include slapping, pinching, verbal abuse, lack of privacy and discrimination [3]. In recent years, evidence from several sub-Saharan African countries, including Tanzania [4, 5], Kenya [6, 7], Nigeria [8] and Ghana [9–11], suggests that mistreatment during childbirth may be a common occurrence, and may be exacerbated by certain characteristics of the woman, including age and HIV status. This work has been groundbreaking to better understand women’s experiences of mistreatment during childbirth, as well as efforts to measure mistreatment that does occur, through observations of labor and childbirth, and follow-up surveys with women. Rominski and colleagues explored justifications for mistreatment during childbirth among midwifery students in Ghana and found that participants rationalized this type of care to help the mother and baby, and that there was no alternative to mistreatment during childbirth in their settings [11]. In Guinea, anecdotal evidence from clinical practice and discussions with women suggests that women are often mistreated during childbirth. This anecdotal evidence has been supplemented by a qualitative study exploring women’s and healthcare providers’ perceptions and experiences of mistreatment during childbirth [12]. Balde and colleagues found that both women and providers reported instances of physical abuse (pinching and slapping), verbal abuse, abandonment and neglect [12]. Women also reported giving birth on the floor of the health facility, and giving birth without the presence of a skilled attendant [12]. However, there is limited understanding of how social norms, acceptability and justification for mistreatment during childbirth influences its occurrence.

This study was conducted to explore and understand how women are treated during childbirth in health facilities, and is part of a multi-country study in Guinea, Ghana, Nigeria and Myanmar [13]. In short, the study is comprised of a qualitative formative phase and a quantitative measurement phase, and aims to better understand how mistreatment during childbirth is occurring, contributing factors, and how to measure its occurrence. In Guinea, this study has been implemented in the prefectures of Mamou and Pita, in the administrative region of Mamou. This region is 300 km from Conakry, with a total regional population of 437,936 inhabitants. The urban center of Mamou is home to 30,982 people and has a regional hospital and 5 health facilities, and Pita has a population of 18,676 people.

This paper presents a qualitative analysis of the acceptability of the mistreatment of women while giving birth, according to women and service providers. Understanding how attitudes and social norms influence how women are treated is an important step to understand why women are mistreated during childbirth and how to prevent it from happening.

### Overview of women’s health in Guinea

Maternal mortality in the Republic of Guinea has reduced over time, but remains an important challenge to improving maternal health. In 2005, maternal mortality accounted for 36% of deaths for women aged 15–49 years [14], while in 2012 it represented 28% of deaths for the same population [15]. Similarly, the maternal mortality ratio (MMR) decreased between 2005 and 2012, from 980 to 724 maternal deaths per 100,000 live births [14, 15], and women now have a lifetime risk of maternal mortality of 1 in 25 [16]. The 2012 Demographic and Health survey showed that across Guinea, 45% of childbirths are carried out with the skilled health workers, 25% with traditional providers, 18% by parents or friends and 7% without any assistance [15]. Violence against women is common in Guinea, with 92% of women aged 15 – 64 reporting ever experiencing violence and 40% experiencing violence in the previous twelve months [17]. The primary drivers of violence against women in Guinea are gender imbalances in decision-making and economics, social tension and the feminization of poverty [18].

Key challenges to improving quality of care include improving the theoretical and technical knowledge of health workers, addressing health workforce shortages and better equipping the health facilities with necessary physical resources. The 2012 Demographic and Health Survey also concluded that to cover the needs of the population of Guinea, 2,263 midwives were needed, however currently only 409 midwives were employed nationally, a shortfall of 82% [15]. Although Ebola virus has affected Guinea since 2014, the study area was minimally affected by the outbreak.

## Methods

### Study sites

The two sites selected for this study (Mamou and Pita) are in the same administrative region. Mamou is an urban location with a regional hospital, and Pita is a peri-urban location with a prefectural (district-level) hospital. This study took place in these health facilities (both provide maternity services), as well as the communities that are within the facility catchment areas. Health indicators in the Mamou region are worsethan at the national level. For example, the maternal mortality ratio at the Regional Hospital of Mamou was 1172/100,000 live births in 2015, and approximately one-third of women in Mamou give birth without any assistance [19]. The mean age of first marriage for women is 17 years, compared to men at 26 years, and the total fertility rate is 5.4 (number of children born per woman), compared to the national fertility rate of 3.8 [15].

### Study participants, recruitment and sampling

Three groups of participants were identified for this study. First, in-depth interviews (IDIs) and focus group discussions (FGDs) were conducted with women of reproductive age (18–49). Inclusion criteria for women of reproductive age is: women with previous experience (previous 1 year for IDIs and previous 5 years for FGDs) of childbirth in a health facility and currently living in the facility catchment area. Community health workers identified women who met the inclusion criteria and helped connect the research assistants in person. FGDs were conducted separately for younger women (18 – 24 years) and older women (25 – 49 years), to ensure that participants all had an opportunity to share their opinions and did not feel societal pressure to defer to their elders. Second, IDIs were conducted with midwives, nurses and doctors working on the maternity ward of the study facilities. Third, IDIs were conducted with facility administrators, such as the medical director or matron-in-charge. Quota sampling was used to achieve a purposive sample without random selection, with specific parameters to enhance variation in the sample. Participants were recruited until the pre-specified sample size was reached, and no new themes emerged from the data (data saturation). All potential participants were invited to participate and provide consent.

### Discussion guides

This study used a qualitative approach to data collection, with semi-structured IDI and FGD guides. The discussion guides were similar among the different groups of participants to allow for comparability of responses, and covered these topics, in the following sequence: (1) story of childbirth; (2) perceptions and experiences of childbirth occurring in health facilities; (3) elements and experiences of mistreatment during childbirth; (4) perceived factors influencing how women are treated during childbirth; (5) acceptability of scenarios of mistreatment during childbirth. Discussion guides were piloted with doctors, midwives and women, and refined during a training workshop for the research team. In order to build a relationship between the interviewer and the participant, each IDI and FGD started with more general questions about the childbirth experience, expectations of care and what supportive care means to them. Then, participants were asked if they, or a friend or family member, experienced anything during their childbirth that made them feel unhappy or uncomfortable. Participants were probed on who was involved in the incident, when and why it happened, and if they felt it was common to be treated in this way. Providers were asked the same questions, as well as whether they had seen or heard of any poor treatment of women during childbirth occurring in their work place. After personal experiences were shared, participants were presented with four scenarios that could be classified as mistreatment during childbirth, based on a systematic review [3] and pilot testing with key stakeholders: (1) a provider pinching or slapping a woman in labor; (2) a provider yelling or shouting at a woman in labor; (3) a provider refusing to help a woman in labor; and (4) forcing a woman to deliver on the floor of the hospital. Participants were asked if the scenario was acceptable, under what circumstances (if any) it would be acceptable, and how they would feel if it happened to them (for women) or their female partner/sister (for men).

### Data collection and management

The research team for this study is a group of medical doctors and sociologists affiliated with Cellule de recherche en santé de la reproduction (CERREGUI). There were 10 data collectors in total, eight women and two men. Before starting data collection, there was a training workshop in Conakry for the research team. During the workshop, the study protocol and discussion guides were discussed in detail and interviewers were trained. All IDI and FGD discussion guides were pre-tested in order to evaluate, improve and adapt the discussion guides for the Guinea context. During data collection, IDIs and FGDs with women were conducted in private, quiet areas in the community, and data collectors were women only. IDIs with providers and administrators were conducted in a private room in the health facility. All participants were contacted once. IDIs and FGDs lasted approximately 60 – 90 min, and participants received a snack and drink to show appreciation for their time. All IDIs and FGDs were audio recorded, and transcribed verbatim from local language (Pular and Malinke), then translated to French by the research team. Data collection and transcription was four months in duration (June to September 2015).

### Data analysis

We used a thematic analysis approach, as described by Braun and Clark [20]. The analysis process started at an analysis workshop for the study teams from Guinea, Ghana and Nigeria. We used the typology of mistreatment during childbirth proposed by Bohren and colleagues [3] to start building the codebook. The codebook was supplemented with codes emerging from the data and from the discussion guides. Coding was conducted manually using Microsoft Word by two researchers from CERREGUI with medical and sociology training, with the support of the research team. Throughout the research process, the researchers considered how their worldview and training may influence their interpretation of the results (reflexivity through discussions with the research team. These discussions started during study design, and continued throughout training workshops, data collection, and a data analysis workshop. Researchers were encouraged to consider how their own experiences of childbirth (or their partners’, family members’, or friends’ experience of childbirth), their training and awareness of the topic may influence their interpretation of results, and how the results may influence their perspectives.

### Ethical and technical approvals

This study was approved by the le comité national d’éthique pour la recherche en santé (National Ethics Committee for Health Research) in Guinea (protocol number: 024/CNERS/15). This study was also approved by the World Health Organization Ethical Review Committee (protocol number: A65880) and the World Health Organization Human Reproduction Programme (HRP) Review Panel on Research Projects (RP2).

## Results

A total of 64 IDIs and 8 FGDs were conducted and are included in this analysis, including 40 IDIs and 8 FGDs with women of reproductive age, 5 IDIs with doctors, 13 IDIs with midwives and 6 IDIs with hospital administrators. Table 1 presents the sociodemographic characteristics of service providers, and Table 2 presents the sociodemographic characteristics of women. Most women in this study were housewives or traders in the informal sector, Muslim and currently married. More than half of women had no formal education and had two or three children. All nurses and midwives were female (typical for Guinea), and most were less than 30 years old. All doctors were male, and most were less than 40 years old.

**Table 1:** Sociodemographic characteristics of participants: healthcare providers and administrators

	Nurse/midwives *n* = 13	Doctors *n* = 05	Administrators *n* = 06
Age (years)			
<30	6	1	0
30–39	3	3	0
40–49	2	0	1
≥ 50	2	1	5
Marital status			
Single	1	0	0
Married	12	5	6
Widowed	0	0	0
Gender			
Female	13	0	0
Male	0	5	6
Years of experience			
0–4	5	2	1
5–9	5	2	0
10–15	1	0	0
15+	2	1	5
Hospital location			
Urban	8	4	3
Peri-urban	5	1	3

**Table 2:** Sociodemographic characteristics of participants: women of reproductive age

	IDIs (*n* = 40)	FGDs (*n* = 8 FGDs^a^)
Age (years)		
<20	10	13
20–24	10	20
25–29	9	21
30–34	4	8
35–39	6	5
≥40	1	2
Marital status		
Single	0	1
Married	39	68
Divorced/Widowed	1	0
Location		
Urban	20	39
Peri-urban	20	30
Rural	0	0
Religion		
Christian	0	7
Muslim	40	62
Education		
None	18	36
Primary	6	11
Secondary	14	19
Tertiary	2	3
Diploma	0	0
Employment		
Business/private sector	0	0
Civil servant	1	0
Hair dresser	0	3
Housewife	7	24
Tailor	5	15
Teacher	3	2
Trader	18	21
Students	6	4
Number of living children		
0–1	12	23
2–3	16	32
4–5	7	11
6+	5	3

^a^8 FGDs conducted with a total of 69 participants (6 FGDs conducted with 9 women, 1 FGD with 8 women and 1 FGD with 7 women)

Exploring women’s and providers’ attitudes towards mistreatment during childbirth is a crucial aspect of understanding why mistreatment occurs and how to prevent it from occurring. When speaking generally about mistreatment during childbirth, women and providers in Guinea felt strongly against such acts. However, when exploring more deeply about the acceptability of acts of mistreatment during childbirth under certain circumstances, opinions are more nuanced and are explored in detail in this analysis. This analysis explores women’s and providers’ acceptability of each of four scenarios about mistreatment during childbirth, as well as the participant’s reaction if they were the victim of such mistreatment.

### Scenario 1: pinching or slapping a woman during labor and childbirth

The majority of women believe that slapping or pinching a woman during labor is not acceptable, because woman in labor are already suffering from pains, anxieties and worries. When women are already agitated from the pains, being slapped or pinched by a provider was perceived to be counterproductive, particularly because the providers are supposed to be helping them.

*Interviewer: When is it* [slapping or pinching] *acceptable?*

*Participant 7* ***:*** *Never acceptable, because all women in childbirth know that contractions are painful…Some women in childbirth do not respect suggested positions, but it does not justify slapping or pinching the woman.* [FGD women, 24 years old, urban]

*Interviewer: If a woman was pinched or slapped by a health worker during her childbirth, would this be acceptable?*

*Participant: It is not acceptable. If I am in [labor] I don’t to be insulted, or pinched, or brutalized. Because if it is my sister, it is not acceptable, so it should be equal for all women.* [IDI female midwife, 32 years old, peri-urban facility]

Rather than using physical force to encourage a woman to cooperate, women suggested that providers should ask the people who accompanied the woman to the facility (e.g.: her mother/mother-in-law, husband, sister or friend) for their assistance to support the woman.

Women who provided justification for pinching or slapping ultimately believed that providers were using physical force in order to save the woman or baby’s life, and approximately one-quarter of women in this study were accepting of pinching or slapping for this reason.

*Participant P5: Why do they slap you? Some are difficult, refuse to obey doctor’s recommendations, not to harm the baby, the doctor can get upset against the woman because he is scared of the baby’s death.* [FGD women, 25 years old, urban]

Women who were accepting of slapping or pinching believed that women’s behaviors during labor and childbirth put their lives or their baby’s lives at risk. These behaviors can be organized into four categories: (1) when the woman refuse to cooperate; (2) when the woman is impolite, rude or insulting to the provider; (3) when the woman crosses her legs during childbirth; and (4) when the woman shouts during labor and childbirth. When women justified pinching or slapping for any reason, they were likely to justify it for more than one circumstance.

*Participant P9: Some women are very difficult. When I went to childbirth at the communal health facility in Conakry, midwives hit a woman very well, it was due to how she delivers, because she was crying badly by saying “woyoyi daddy, mummy, help me, help me,” … It’s because she was difficult, that is why she was hit. She was not stopping shouting. She was hit due to her behavior.*

*Participant P3: …it is because it is sure if she does not keep quiet, something bad will happen to the baby or the mother. At that time, she [should] be hit.*

*Participant P2: If you go there to make a row, they will hit you. But if you go there not to be hit, they will not hit you.*

*Participant P5: Ah! If you do not keep quiet, they will slap you.* [FGD women, urban]

Slapping or pinching a woman during childbirth was sometimes acceptable if she is uncooperative, “difficult”, or refuses to collaborate with the providers. When women are uncooperative, slapping or pinching could be used to stigmatize the woman’s response and encourage her to obey the providers’ instructions.

*Participant: There are difficult women, some of us stand, bend, kneel or hop. They hit you, or bend on you, they tell you, “you have to lie down here to avoid issues to the baby.” At that time, if they insult me or hit it is acceptable because it is to help me.* [FGD women, 24 years old, peri-urban]

Following the providers’ instructions was perceived to facilitate a favorable and pleasant outcome during childbirth. Women often used expressions like “refuse to keep quiet”, “a difficult woman”, “refuse to cooperate” to underline the necessity of absolute submission of the woman to the provider during childbirth. These women were viewed as not understanding or adhering to the instructions given by the providers. Women also felt that when women have poor attitudes during their interactions with providers, or if she insults the provider, then the provider “*ought to slap*” the woman.

*Participant P10: If you go there and you show impoliteness, you do not apologize, they will leave you there, to do what you want until the delivery time… But it is not normal for a doctor to pinch or slap because at that time she is not controlling herself. If you show impoliteness to a doctor when he is assisting you, at that time he can hit you, he does not have any sin towards you.*

*Participant P8: It’s when those who childbirth use harsh language or they are difficult and when they prevent them to do their work. So they get upset, that is why they do things like that. If not, if you show them you are wise, they will really take care of you. If you show them impoliteness, they will badly take care of you, and it is not their will, it’s up to you.* [FGD women, urban]

*Participant P10: there are others, if they go to childbirth, they are very difficult to control, they can shout or insult. I saw some women insulting their husbands by telling them they are responsible for what they are facing. All this can irritate people who are beside you. It can push him/her to slap you. All this, you are the one who creates this.* [FGD women, 27 years old, urban]

Similarly, when women cry out “excessively” due to labor pains, some women believed that slapping or pinching a woman are acceptable means by which to keep her quiet. Their rationale is that a woman’s cries may irritate the provider, and that slapping her will help the woman to focus and deliver silently.

During labor, some women cross their legs either to help cope with pain or to maintain dignity and privacy. When this happens, some younger women considered it acceptable for providers to slap or pinch the woman as a corrective measure to encourage the woman to open her legs. These women believed that slapping a woman who closed her legs is acceptable “because it is to save the baby”. However, older women (>25 years old) and providers did not share this view.

*Participant P5: It’s when the baby is getting out, because there are some women who tighten the legs, if the doctor does not have any solution he will hit the legs at that time, it is acceptable at that time it’s to help you.*

*Participant P4, 22 years old: During delivery time if the woman crosses her legs or does some movements which can harm the baby, I can accept.* [FGD women, peri-urban]

In contrast, providers did not disclose any situations that they believed were acceptable to slap or pinch a woman during childbirth.

### Scenario 2: yelling or shouting at a woman during labor and childbirth

The majority of women and health workers believe that it is not acceptable for a health worker to shout at a woman during childbirth, and preferred that service providers talk gently to the woman to guide her through labor without shouting. Shouting or yelling disturbed the woman and some women felt that it can negatively influence her labor progress. However, shouting happens frequently and may be considered “normal” behavior:

*Participant R1: Yes, it is normal, if you are not quiet, they should shout on you. But if you are quiet they don’t shout on you.*

*Participant R3: It is normal in two cases and if it is to save the baby, they tell you to “do this or do you want to kill your baby?” They should shout on you telling you to act like that, do like this in order not to kill your baby, you do it gently, and don’t go beyond.…*

*Participant R8: In one way shouting is good. There are women, when they start labor, they lose reference marks; to shout on those ones is good.*

*Participant R5: There are women, they arrive, they are suffering, then they tell them to act gently, they shout on them to reduce their agitation…*

*Participant R3: In two cases it’s normal, one if it is to save the baby, they tell you to this or would you like to kill the baby, they ought to yell at you by saying do like this, for not killing the baby they do it slowly without going beyond the norms.*

*Participant R2: Yelling at you is good if it is to help you for not delivering in bad position or preventing losing the baby.* [FGD women, urban]

Approximately half of the women in this study gave scenarios in which shouting may be acceptable, whereas a small minority of health workers believed that shouting was acceptable under any circumstance. For both groups of participants, the most common scenarios where shouting would be acceptable were when the woman “does something wrong”, is disobedient, “not calm”, due to “excessive shouting” of the woman, or to save the baby’s life. Health workers used shouting in an attempt to reduce the woman’s agitation and encourage her to cooperate.

*Interviewer: When would shouting be acceptable?*

*Participant R9: If it comes from the behavior of the woman, yes, but if without any reason, the worker shouts on you, it is not acceptable…*

*Participant R7, 24 years old: In fact, if it comes from the woman it is acceptable, but if the woman didn’t do anything bad, then it is not acceptable.* [FGD women, urban]

*Interviewer: If a woman was yelled or shouted at by a health worker during her childbirth, would this be acceptable?*

*Participant: You can effectively yell, but don’t hit. When you can yell, is only if the woman closes her legs when she is in complete [cervical] dilation]. So she won’t kill her baby, you can yell at her, or even bring people to hold her. Because, there if you don’t do that, you are in risks of killing the baby.* [IDI male doctor, 52 years old, peri-urban facility]

Women were reproached by health workers for being disobedient, and some women and one health worker believed that shouting was then acceptable “if you refuse to obey,” “refuse to follow the doctor’s midwife’s instructions,” or if the woman makes “errors”.

*Participant: Acceptable if you want to save the baby or the mother. She does some actions and now you can’t slap her or hit her, you yell without noticing in that case it is acceptable if not you have to advise her slowly she will understand.* [IDI female midwife/nurse, 28 years old, peri-urban facility]

*Participant R6: It’s when she is not calm and they have already told her to be calm, they can yell at her…*

*Participant R7: It’s when they tell her to be calm and she refuses, there they can yell at her to prevent issues for them.* [FGD women, peri-urban]

However, neither women nor health workers discussed how poor communication between the health worker and woman, or a woman’s lack of knowledge of the process of labor and childbirth, could contribute to what is perceived as a woman’s disobedience.

Some women also suggested that a provider could shout at a woman when the woman is making “too much noise” during labor. They believed that when women cried out, it disturbed the rest of the hospital and having a health worker yell at her would help to calm her down.

*Participant: It’s when the women cry more or refuse to be calm. For that you can shout just to make her calm.* [IDI woman, 26 years old, peri-urban]

The final condition of acceptability of a health worker yelling at a woman is if the woman closes her legs when the baby’s head is coming out or during the delivery of the placenta.

*Participant R2: It is acceptable if it is to save my baby and myself that they shouted on me.*

*Participant R5: It is normal because if you tighten your legs on the baby, you’ll suffer and make your baby suffer.*

*Participant R3: It is normal in two folds. First it is at the time the baby is engaged, second at the moment they take out the placenta. We hear that if you are not quiet at this time, if the placenta remains there you will bleed. So shouting is normal.* [FGD women, peri-urban]

*Participant: You can effectively yell, but don’t hit. Where you can yell, it occurs only if the woman closes her legs, she is in complete dilatation, for not killing her baby, you can yell at her even bring people to hold her down…because, if you don’t do that, you are at risk of killing the baby. But sometimes you are not responsible of what you are doing. We mustn’t hit, yell in normal conditions, but if the woman is in a complete dilatation you will yell.* [IDI male doctor, 52 years old, peri-urban facility]

In this circumstance, yelling is considered appropriate in order to prevent the baby’s death or the woman bleeding during childbirth.

### Scenario 3: refusing to help woman during labor

Participants were asked about their acceptability of a provider refusing to help a woman during labor. All respondents (women and service providers) recognize that it is unacceptable for a health worker to refuse to assist a woman during childbirth. One women expressed that “*if you go to hospital you ought to be helped”* [FGD women, 43 years old, peri-urban]*.* Another woman stated that the providers already received a salary to provide services; thus, the providers had a moral obligation to take care of the women:

*Participant: No, no one will accept it. You go to hospital and they take your money without assisting you. No, it is not good. It must not be, because if they take your money they ought to take care of you, don’t they? They take care of you until you recover.* [IDI woman, 35 years old, peri-urban]

However, women explained that providers often refused to assist them during childbirth, because they did not pay the requested “informal payment”. In Guinea, maternity services have been free of charge since 2011. Informal payments to a health worker for her services was not unacceptable to women; rather, it was paying the health worker as a prior condition to care that women found unfathomable.

*Participant P8: It’s when she delivers and she (midwife) is beside you ought to give her at least something even though she does not request*.[FGD women, 30 years old, peri-urban]

Women expressed that if they were satisfied with their care, then they would often willingly offer a small gift or payment to the health worker — a culturally acceptable expression of their gratitude.

### Scenario 4: forcing a woman to deliver on the floor of the hospital

Participants were asked if providers forcing a woman to deliver on the floor of the delivery room was acceptable. This refers to the providers demanding that the woman gets down off the hospital bed to deliver on the floor. Almost all women considered this to be a grave form of mistreatment that caused an undue amount of shame on the woman. They believed that a main reason that women go to the hospital for childbirth is to give birth in a hospital bed; therefore, not allowing this to happen is a serious concern with lasting impact.

*Participant: It is not acceptable. Even if the woman who delivers will not accept it, she would prefer to go home and deliver herself, even though she will die.* [IDI woman, 23 years old, peri-urban]

A small minority of women suggested that there could be situations where it was acceptable for women to be forced to deliver on the floor. The first scenario is if the woman refuses to calm down, be quiet and stay still, then the provider may force her onto the floor to prevent her from falling.

*Participant: During contractions, they might go on the floor to avoid falling. If she is doing too many movements which are not good, we send her to the floor, it when she does too much bad movements.* [IDI woman, 23 years old, peri-urban]

*R: It is when they ask her to keep quiet so that she does not fall down and she refuses. There, they can hold her down and put something on the floor and force her down from the table to lie there.* [IDI woman, 18 years old, peri-urban]

Second, if the woman prefers to give birth on the floor then she should be allowed to, for example if she were uncomfortable on the bed or the bed was too narrow.

*Interviewer: If a health worker forced a woman down from the table during childbirth would this be acceptable?*

*Participant 1: It must not be, but there are others if they go there to childbirth, they do not stay in bed to childbirth, they sit on the floor and childbirth or lie down on the floor and childbirth. It is what they prefer.* [FGD woman, peri-urban]

However, women agreed that this should only happen if the hospital was clean, which they did not expect to happen: “*I will not be happy at all because the hospital is not clean to hold someone like that*” [FGD women, peri-urban]

Providers agreed that forcing a woman to deliver on the floor was inappropriate and should not happen. However, several providers described that some women ask them to move the mattress onto the floor for them to give birth. In this circumstance, the midwife would try to accommodate the woman’s request, but would not force the woman to do so.

*R: It is acceptable if the woman decides to give birth on the floor. But there too, you do not let her give birth on the floor directly, you get down the mattress from the bed and she gives birth there.* [IDI nurse/midwife, 32 years old, peri-urban]

Providers also noted that women were sometimes forced to deliver on the floor of the hospital because all beds were full: “*When the delivery room is full, and all beds are busy and another woman comes to childbirth, we don’t have anywhere to put her”* [IDI nurse, 48 years old, urban].

### Comparing the acceptability of circumstances for mistreatment during childbirth

There are similarities of when the four scenarios of mistreatment during childbirth would be acceptable to the women and providers in this study. Table 3 presents a comparison of the circumstances where mistreatment during childbirth may be acceptable. Slapping and yelling at a woman who considered acceptable to punish a woman for several of the same “indiscretions”, including women who crossed her legs during the birth, or women who were uncooperative, disobedient, or making too much noise. Interestingly, a provider slapping a woman was also considered acceptable punishment for a woman being rude, impolite or insulting to a provider, yelling at the woman was not considered acceptable behavior in this circumstance. Having the woman give birth on the floor of the health facility was another form of punishment for women being disobedient, uncooperative or making too much noise during labor. Additionally, giving birth on the floor was considered acceptable for other health system constraints, such as a lack of bed space or if the woman did not have contractions, and thus did not need to be on the delivery bed.

**Table 3:** Comparison of women’s and providers’ perspectives on the acceptability of circumstances for mistreatment during childbirth

Circumstance	To slap	To yell	Refuse to help	Deliver on floor
Woman is uncooperative or disobedient	✓	✓	–	✓
Woman is impolite, rude or insulting to the provider	✓	–	–	–
Woman crosses her legs during delivery	✓	✓	–	–
Woman is making “too much noise” during labor	✓	✓	–	✓
Wish expressed by a woman in labor	–	–	–	✓
Prevent a woman from falling off the table	–	–	–	✓
If the woman does not have contractions	–	–	–	✓
No more bed space	–	–	–	✓

In particular, there are clear similarities between the circumstances acceptable to slap, pinch or shout at a woman, including if the woman is uncooperative, disobedient, crosses her legs, or is making “too much noise”.

### Reactions of the participants to the four scenarios

Both women and female providers in this study were asked how they would respond if they were on the receiving end of the four scenarios discussed. Male providers were asked how they would respond if their female partner or sister were on the receiving end of one of the four scenarios. Women’s and providers’ reactions to slapping, pinching and yelling scenarios were similar; although yelling was slightly more acceptable than pinching or slapping.

Almost all women reported that they would feel deeply unhappy and angry. For a minority of women, this dissatisfaction would turn into acceptance and forgiveness. Women who believed that they would forgive the provider for their violent gestures believed that they themselves were to blame for inappropriate behavior during labor, which caused the provider to react. These women believed that the provider acted to save the baby’s life: “*if they yell at me, I will not get upset because it’s in my interest*” [FGD woman, urban].

*Interviewer: How would you feel if it happens to you?*

*Participant P10: If a service provider slaps me during childbirth, at that time I will say he has not helped me. But after delivery in good conditions, when I see my baby lying and crying, I would be happy and I will forgive him because if he didn’t guide me, I wouldn’t keep quiet and I will lose my baby*. [FGD women, 24 years old, urban]

However, most women and providers believed that her unhappiness would influence her next actions and decisions, including to influence her care-seeking behaviors, cause her to complain to a supervisor, or to enact revenge against the offending provider. Many women believed that they would not choose to deliver in that hospital again, in order to avoid poor treatment in the future, and some women even threatened to deliver at home. Health workers agreed that they would not recommend for women who had been physically abused to return to the same hospital again.

*P7: Will never go there anymore, we will get upset and if we see someone going there, we will tell the person not to go to that hospital, because they are many hospitals she can go somewhere else but not there.* [FGD women, 22 years old, peri-urban]

*P2: If they slap me, I will not go back there. I am suffering I would concentrate on myself and childbirth at home.* [FGD women, 26 years old, urban]

Second, some women believed that they would be so angry at the provider for slapping or pinching them, that they will physically attack the offending health worker after their delivery. These women felt that this would be appropriate “payback”: “*if a service provider does it to me I will wait till I finish and we will fight*” [IDI woman, 39 years old, urban]. A minority of health workers agreed that they would “payback” the offending health worker.

A majority of providers but a minority of women explained that they would complain to the provider’s supervisor, in hopes that the supervisor will take action to prevent this from happening again. Both women and providers believed it would be best to wait until after the delivery was finished, so that a complaint did not further influence their treatment.

Reactions to providers refusing to help a woman were varied. First, many women felt that their only possible reaction was to submit to the situation and “*the best thing is to give yourself to God will*” [FGD women, urban]. Many women felt that they would be very unhappy, but that there was nothing they could do to change the situation. In contrast, providers felt they would be “beyond anger” and believed that they would talk directly to the provider denying treatment. Some women and almost all providers believed they would complain to the hospital supervisors, believing that refusing to help a woman in labor is “criminal” and such behavior should be punished. In the future, both women and providers would not want to be cared for by the same provider, and may seek care in other hospitals.

Reactions to forcing a woman to deliver on the floor depended on the situation. When women were forced from the bed because of slow contractions, they would follow the instructions of the provider, albeit reluctantly as it was considered a shameful request. More than half of women believed that although they were unhappy, they would not react further. However, providers stated that they would “refuse to get down” [IDI nurse/midwife, 57 years old, peri-urban], demand an explanation from the provider in order to understand why this happened, and complain to the supervisor. Some women and providers felt that they would either leave the hospital immediately to finish giving birth at home, or never go back to that hospital for childbirth in the future.

*Participant: I will badly react. We don’t have to force a woman down from the table to make her deliver on the floor. It is not good. I will leave the hospital and go home to childbirth. Next time I will stay at home.* [IDI nurse/midwife, 28 years old, urban]

*R:It is not acceptable, she prefers to go back home and deliver there by herself, even if she will die, she prefers that than delivering on the floor in hospital.* [IDI woman, 23 years old, peri-urban]

## Conclusions

We explored the acceptability of four scenarios of mistreatment during childbirth: physical abuse, verbal abuse, refusal to assist a woman in labor, and forcing a woman to deliver on the floor of the delivery room. For each scenario, we explored the circumstances under which the scenario would be acceptable (if any) and why these circumstances were acceptable. This study demonstrates that in Guinea, both women and service providers are accepting of mistreatment during childbirth under certain circumstances. Midwives and doctors may use abusive techniques to get women to cooperate, and paradoxically some women accept such mistreatment if they believe it will benefit their health or their baby’s health. These results are important because they highlight the types of mistreatment that women are subjected to during childbirth in the hospital, and the social norms regarding whether such treatment should be acceptable or not.

Furthermore, social norms regarding violence and how women are treated may be deeply held in Guinea. According to the 2012 DHS, the majority of women (92%) and men (66%) believe that a man beating his wife is acceptable under at least one condition, including burning the food, arguing with him, leaving the house without telling him, neglecting the children, or refusing sexual intercourse [15]. This justification for violence among the majority of women in Guinea is a reflection of the social construct in which they live. Findings from this study are analogous and suggest that women and healthcare providers believe that mistreatment is justifiable under certain circumstances, such as when women cry out or are noncompliant with providers’ demands. Both women and providers believed that slapping or yelling at a woman was an appropriate response to a woman’s disobedience while in the health facility for childbirth. In this context, women were expected to always obey the requests of the healthcare providers; their own needs and preferences, such as crying out in pain or delivering in a non-supine position, were often ignored. Societal hierarchies in Guinea may systematically disempower women, and could be an explanation for why shameful acts of mistreatment during childbirth are not denounced and perpetrators are not held accountable. Therefore, women’s acceptance of mistreatment during childbirth by service providers may be justified within the context of violence in Guinea.

The capacity of the health system in Guinea is limited and faces several serious challenges, including inadequate numbers of qualified health workers, and weak infrastructure, health information systems, logistics, surveillance, and drug supplies. Minimal investment in health systems, both from the government and international aid, has led to challenges with resiliency, compounded by the 2014–2015 Ebola outbreak. Weak health systems have limited capacity to cope or improve quality of care. In the case of mistreatment during childbirth in Guinea, Balde and colleagues found that insufficient drugs, equipment and physical infrastructure contribute to a stressful work environment and encourages providers to express frustrations at women [12]. Furthermore, health worker shortages have led to women being mismanaged during labor, as there are not enough staff to provide quality care [12]. Women corroborated these views, and believed that mistreatment occurred because health workers were poorly trained and overworked [12]. Similarly, Rominski and colleagues and Moyer and colleagues highlight that mistreatment during childbirth is engrained in the health system, beginning during midwifery training where trainees are exposed to mistreatment and a lack of accountability for such actions [10, 11].

Given the pervasive nature of mistreatment during childbirth, any approach to preventing and eliminating mistreatment during childbirth must consider these important contextual and social norms and develop a comprehensive intervention that addresses root causes. Structuring the midwifery, nursing and medical curriculum to prioritize the importance psychosocial elements of maternity care, such as labor companionship, empathy, compassion, respect, autonomy and choice, would be an essential component. As a starting point, the results from this study will be shared with the Ministry of Health, WHO, hospitals, and communities. We aim to widely disseminate the results both nationally and internationally through our publications and dissemination workshops. We will work with policy-makers to increase their awareness of mistreatment during childbirth in the Guinea context and help them to develop policies to prevent mistreatment from occurring. Future research could explore the experiences of mistreatment during childbirth in other areas of Guinea to explore if our results from this study are transferable. Furthermore, this study should be used to inform the development of tools to measure the occurrence of mistreatment during childbirth in facilities, and also to develop intervention or programs to prevent mistreatment from occurring.

### Limitations and strengths

This study has some weaknesses and some strengths. Although the study sites were minimally affected by Ebola, during the epidemic many communities associated health professionals or discussions about health with Ebola. Therefore, recruiting participants was sometimes challenging, as some husbands refused to allow their wives to participate in the study. However, the study team worked to dispel these fears by reassuring potential participants that this study was related to childbirth, and would not put them at risk of Ebola. Interviews were often conducted in local languages (Pular and Malinke), and transcription from local language to French for analysis was challenging and time-consuming. This study was conducted in two towns in one prefecture of Guinea. However, we believe that the results may be transferable to other prefectures in Guinea because women across the country often face the same social contexts and realities. Furthermore, Mamou is an urban area that attracts people to move from different areas of Guinea; therefore their experiences may also be reflected in our study. This is the first study on mistreatment of women during childbirth in Guinea. Our research team consists of both sociologists and medical professionals, which strengthened our ability to interpret the results. Additionally, we were able to share the experiences of mistreatment during childbirth in the Guinea context with our colleagues working on the same project in Ghana and Nigeria. The interdisciplinary structure of the study team and analysis approach is an asset that has helped to overcome any challenges faced.

### Research implications

This study suggests that women in Guinea experience mistreatment during childbirth, and that they may be accepting of this mistreatment under certain circumstances. These circumstances mostly relate to if the mistreatment is perpetrated with the belief that it will save the life of the mother or her baby. Moving forward, we encourage the Ministry of Health, in collaboration with WHO, to use these results to integrate the prevention of the mistreatment of women during childbirth into the national maternal health strategy. This requires efforts to change the behavior of the providers, which may be achieved through training workshops, sensitization programs and re-organization of training curriculum for medical, nursing and midwifery schools.

## Abbreviations

CERREGUI: Cellule de recherche en la santé de la reproduction en Guinée
DHS: Demographic and Health Survey
FGDs: Focus group discussions
HRP: World Health Organization Human Reproduction Programme
IDIs: In-depth interviews
LMICs: Low- and middle-income countries
MDGs: Millenium Development Goals
MMR: Maternal mortality ratio
RP2: Research Project Review Panel
